# Architecture-Guided Fluid Flow Directs Renal Biomineralization

**DOI:** 10.1038/s41598-018-30717-x

**Published:** 2018-09-21

**Authors:** Sunita P. Ho, Ling Chen, Frances I. Allen, Ryan S. Hsi, Alex R. Shimotake, Scott V. Wiener, Misun Kang, Andrew M. Minor, Marshall L. Stoller

**Affiliations:** 1 Division of Biomaterials and Bioengineering, School of Dentistry, University of California San Francisco, San Francisco, CA 94143 USA; 2 Department of Materials Science and Engineering, University of California, Berkeley, CA 94720 USA; 3 National Center for Electron Microscopy, Molecular Foundry, Lawrence Berkeley National Laboratory, Berkeley, CA 94720 USA; 4 Department of Urologic Surgery, School of Medicine, Vanderbilt University, Nashville, TN 37232 USA; 5 Department of Urology, School of Medicine, University of California San Francisco, San Francisco, CA 94143 USA

## Abstract

Nephrocalcinosis often begins on a calcium phosphate deposit, at the tip of the medullo-papillary complex (MPC) known as Randall’s plaque (RP). Contextualizing proximally observed biominerals within the MPC has led us to postulate a mechanobiological switch that can trigger interstitial biomineralization at the MPC tip, remote from the intratubular biominerals. Micro X-ray computed tomography scans of human MPCs correlated with transmission and scanning electron micrographs, and X-ray energy dispersive spectrometry demonstrated novel findings about anatomically-specific biominerals. An abundance of proximal intratubular biominerals were associated with emergence of distal interstitial RP. The fundamental architecture of the MPC and mineral densities at the proximal and distal locations of the MPC differed markedly. A predominance of plate-like minerals or radially oriented plate-like crystallites within spheroidal minerals in the proximal intratubular locations, and core-shell type crystallites within spheroidal minerals in distal interstitial locations were observed. Based on the MPC anatomic location of structure-specific biominerals, a biological switch within the mineral-free zone occurring between the proximal and distal locations is postulated. The “on” and “off” switch is dependent on changes in the pressure differential resulting from changes in tubule diameters; the “Venturi effect” changes the “circumferential strain” and culminates in interstitial crystal deposits in the distal tubule wall in response to proximal tubular obstruction. These distal interstitial mineralizations can emerge into the collecting system of the kidney linking nephrocalcinosis with nephrolithiasis.

## Introduction

Form and function are intimately related. Organ function is dependent on the form of the organ, the size of the organism, and the environment in which it resides. The fundamental functional unit responsible for blood filtration across most organisms is conserved^1^. One such fundamental unit in the human kidney is the paraboloid shaped medullo-papillary complex (MPC) including the renal papilla. This study will “assemble” a cascade of fluid flow processes within the MPC from multiscale biomechanics and mechanobiology perspectives. It argues that the cascade of flow processes is regulated by both the form and intrinsic architecture of the human MPC. Evidence about the effect of form and intrinsic architecture on biomineral formation within the MPC will be extracted through multiscale characterization using high resolution correlative microscopy and materials science approaches.

Nature’s engineered paraboloid shaped MPC of the kidney is predisposed to form biominerals when evaluated from a fluid dynamics perspective. Nanoparticles aggregate to form micro- to macro-meter sized larger concretions subsequently building fertile nidi on which clinically detectable kidney stones form. This multi-length scale anatomically-specific biomineralization was eloquently stated by Jean Oliver as spanning the “kidney to intracellular microsomes”, who went on to say that “we continue on our imagination sparked by physicists through molecules and atoms, electrons and lesser particles and ultimately to the event”^2^. Obstructing kidney stones have afflicted humans for thousands of years. Kidney stones affect one out of eleven Americans with marginal effective prevention strategies or cure, and medical intervention is limited to management. Contemporary dogma holds that the distally forming biominerals in the human MPC described by Randall serve as a “concrete” bed on which stones form at the tip of the MPC^3^. Many have attempted to identify the origins of biominerals within the MPC, and kidney stones in particular, in an exhaustive manner. Although metabolomics, metallomics, and proteomics-related information regarding stone pathogenesis continue to be gathered^4^, Randall’s plaque formation commonly observed by clinicians at the tip of the MPC per se remains poorly understood. Several studies over the decades have related plaque formation to changes in environmental stimuli, metabolic disturbances, and breakdown of extracellular matrix proteins^5–13^. In this study, it is proposed that Randall’s plaque formation originates from a culmination of several upstream events, proximal to rather than at the tip of the MPC in the human kidney. To appreciate these upstream events in an anatomically and functionally accurate way, the MPC is described as biomechanical and biological continua. By virtue of its paraboloid shape and intrinsic architecture containing several short and long interconnected U-tubes, it seems plausible that mineral formation may begin within the proximal MPC. These processes could signal cascades of biomechanical and biochemical events that culminate and form grossly observed Randall’s plaque. Results from high resolution correlative microscopy will be contextualized by coupling the anatomy-specific mineral formations to the biomechanical and biochemical events unique to the form and function of the MPC. Higher resolution maps of the MPC obtained using electron microscopy and micro-X-ray computed tomography (micro-XCT), and correlative multiscale maps will demonstrate evidence to confirm the statement made by Randall himself in the 1930’s that “urinary calculus is once a symptom of a deeper underlying pathology”^14^ albeit this observation was made with a naked eye.

Previous studies have used advanced imaging modalities to characterize biopsies of the tip of MPC to gather insights into the origins of Randall’s plaque. Nanoscale mineral deposits in the interstitium of the MPC tip are often thought to originate from the basement membrane of the thin loops of Henle^15^. It has been proposed that detectable individual spherical deposits of apatite as small as 50 nm in the interstitial matrix are the beginnings of the plaque development that Randall had observed almost seven decades ago^14,15^. In this study, “deeper underlying pathology” as phrased by Randall will be identified by illustrating the presence of mineral within the proximal region of the MPC. It is for this reason, that the medulla of the MPC is included in this study; this investigation is not limited to the tip of the MPC. A quantitative correlative microscopy approach at multiple length scales will enable contextualizing and thereby postulating the cause for initial upstream events leading to subsequent downstream mineral formations.

## Results

Definitions of words used in the following text to illustrate and discuss results are as follows. Nephrons and vasa recta within the MPC are described as tubules. The open channel within a tubule is defined as the lumen. Intratubular is defined as inside the tubule. Interstitial is defined as the organic matter of the MPC other than that which is occupied by the tubules.

### Insights into anatomically-specific biominerals at multiple length-scales of the MPC: extracting contextual information by correlating data spaces from patient care X-ray computed tomography to high resolution micro X-ray computed tomography with anatomically-specific micro- and nanostructures of biominerals as seen in using scanning and transmission electron microscopy techniques

**Figure 1:**
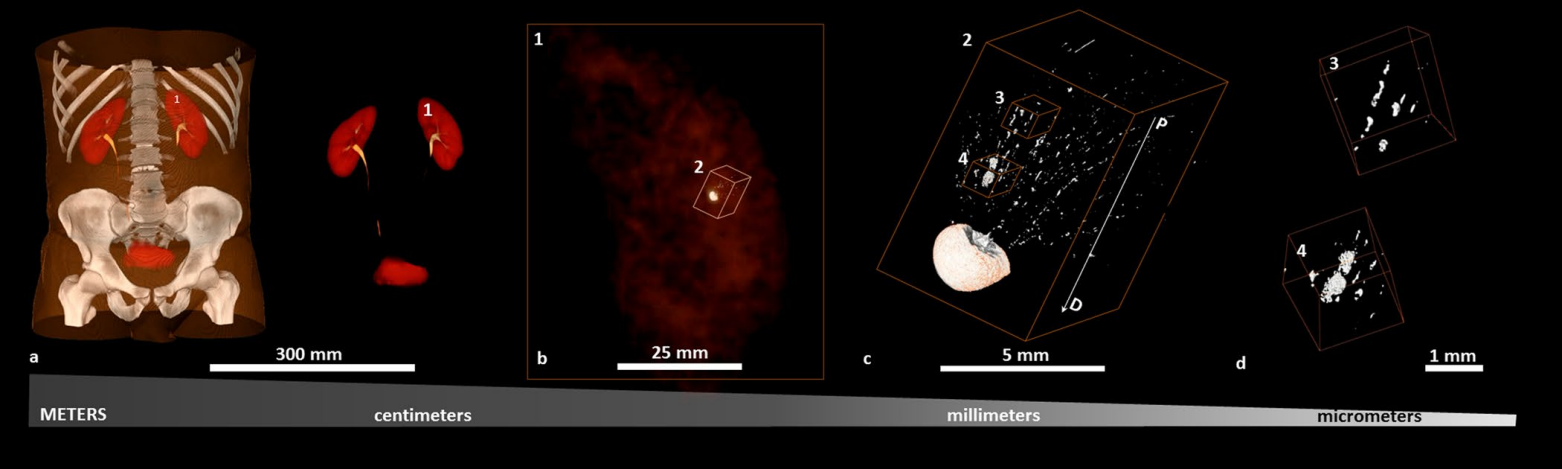
Visualization of reconstructed volumes of interest from a clinical X-ray computed tomography (XCT) to a high resolution *ex vivo* micro-XCT illustrates a hierarchical structure across multiple length-scales. Anatomical location of kidneys within a human torso and digitally isolated kidneys, ureters, and bladder are shown (**a**). Kidney 1 shown in a and b contains a stone attached to the tip of medullo-papillary complex (MPC) (2). The isolated MPC from the kidney (**c**) illustrates biominerals in the proximal (P) and distal (D) regions of the renal parenchyma in addition to the urinary tract stone. Regions 3 and 4 (**d**), respectively, highlight intratubular and interstitial mineral formations.

**Figure 2:**
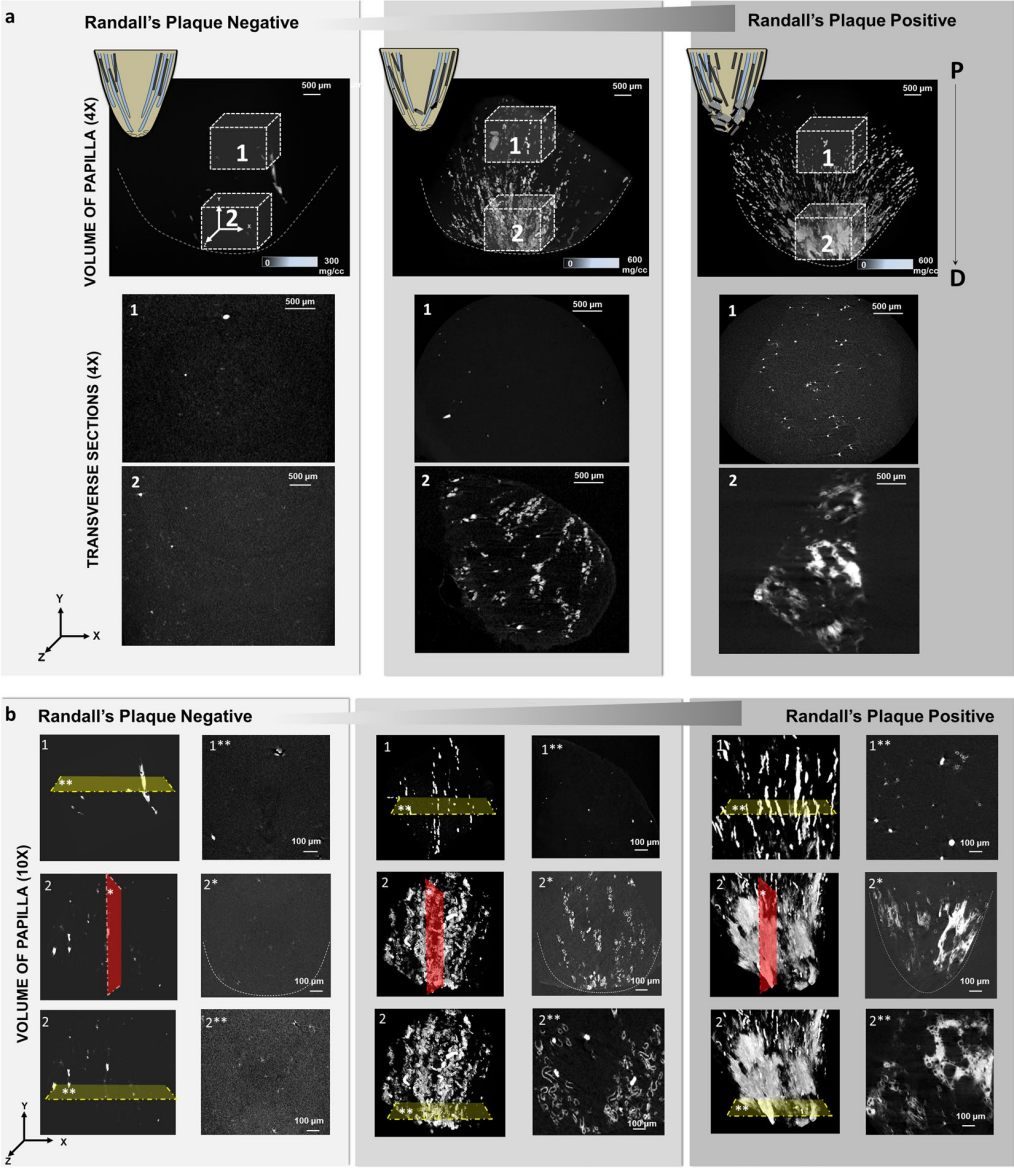
Digitally reconstructed volumes from micro-XCT of various medullo-papillary complexes illustrate distribution of biominerals. Schematic of the MPC is illustrated in the far top left of each column of 2a with proximal occluded tubules (black streaks) representing a complex without Randall’s plaque progressing to interstitial mineralization in the distal region depicted as mineralized blocks, leading to visibly apparent Randall’s plaque positive specimen (endoscopically appreciated at the tip of papilla). Micro-XCT images of minerals within MPC from normal through clinically detectable states containing Randall’s plaque at 4X (**a**) and 10X (**b**) are illustrated. (**a**) Regions 1 and 2 illustrate proximally and distally located volumes within respective complexes. Mineral density scale bars are provided in the bottom right corner of images in the top row. Representative transverse sections highlight mineral within the proximal tubule, distal luminal walls, and interstitium in the clinically detectable complex containing Randall’s plaque. (**b**) Representative transverse (double asterisks, yellow sectioning planes) and longitudinal (single asterisk, red sectioning plane) sections highlight mineral within the proximal tubule in the normal complex compared to those observed in the clinically detectable complex. Note the increase in volume fraction of mineral located proximally and distally within the clinically detectable MPC. P: Proximal; D: distal.

**Figure 3:**
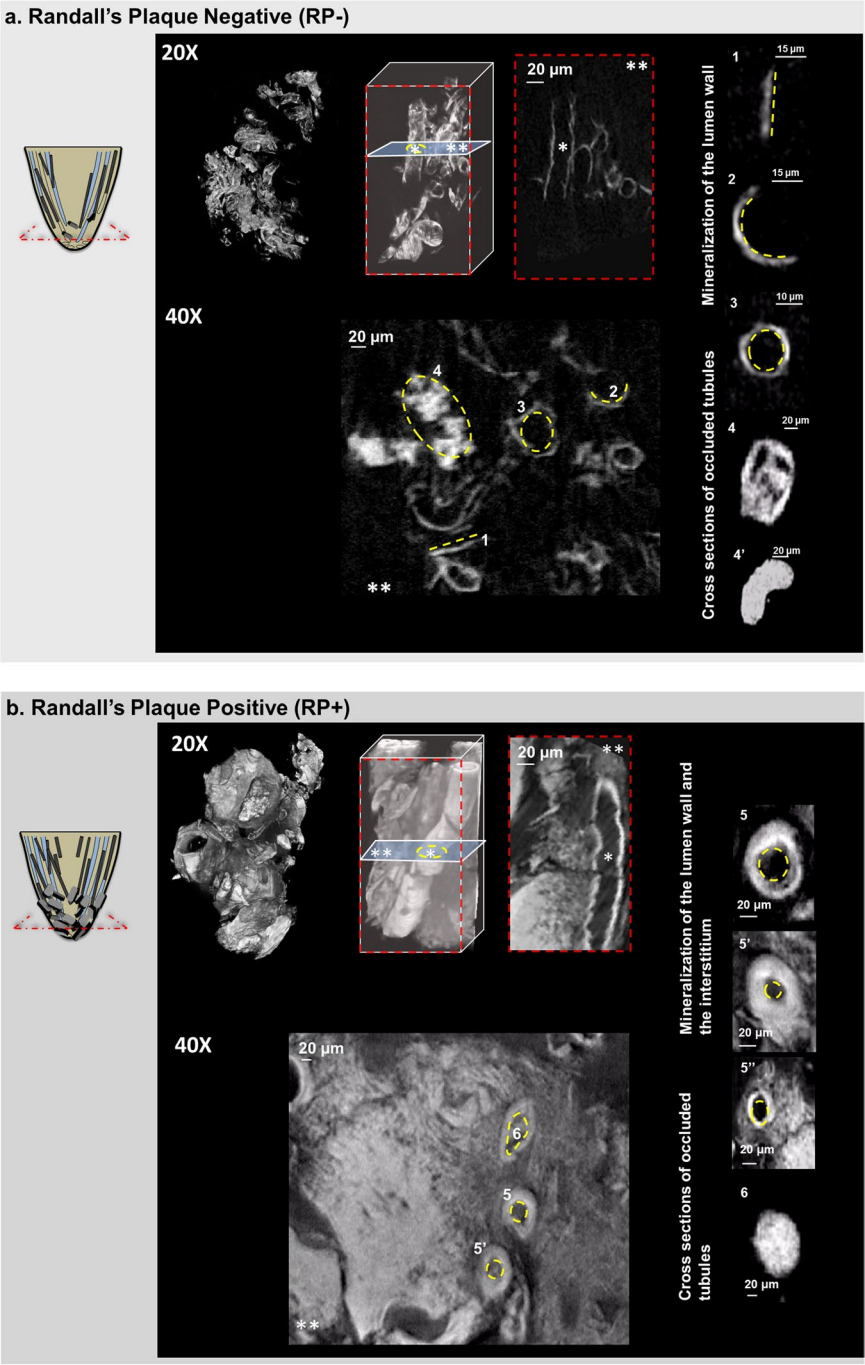
Digitally reconstructed volumes from micro-XCT of the distal region from the medullo-papillary complex with and without clinically detectable Randall’s plaque are shown in **a** and **b**. Schematic of the MPC are illustrated in the far left of **a** and **b** with proximal occluded tubules that are characteristic of a Randall’s plaque negative specimen (**a**, dark streaks) progressing to interstitial mineralization in the distal region depicted as mineralized blocks and ultimately mature into a visibly apparent Randall’s plaque positive specimen (**b**). Micro-XCT images reveal plausible progression (**a**) highlighting partial (**a**,1–3) to complete (**a**,4–4′) mineralization of the luminal wall. However, in a distal region containing Randall’s plaque (**b**), severe mineralization within the interstitium and its progression into collecting ducts is shown (**b**,5–6). Collectively, subfigures 1–4′ and 5–6 provide insights into the progression of biomineralization within the distal region of the renal MPC. Volumes at 20X magnification in (**a**,**b**) depict calcified tubules within the blue dashed rectangles. Longitudinal and transverse sections in (**a**,**b**) illustrate respective cross sections (longitudinal (*) at 20X and transverse sections (**) at 40X). Progression of mineralization within the luminal wall is highlighted with dashed yellow lines. Disease progression (40X) in **b** is illustrated by mineralization of both tubule walls and interstitium (5, 6; yellow dotted circles) and in the extreme case with an occluded tubule (8,8′). Regions 4′ and 6 correspond to cross sections of the same tubule, but from different virtual sections.

**Figure 4:**
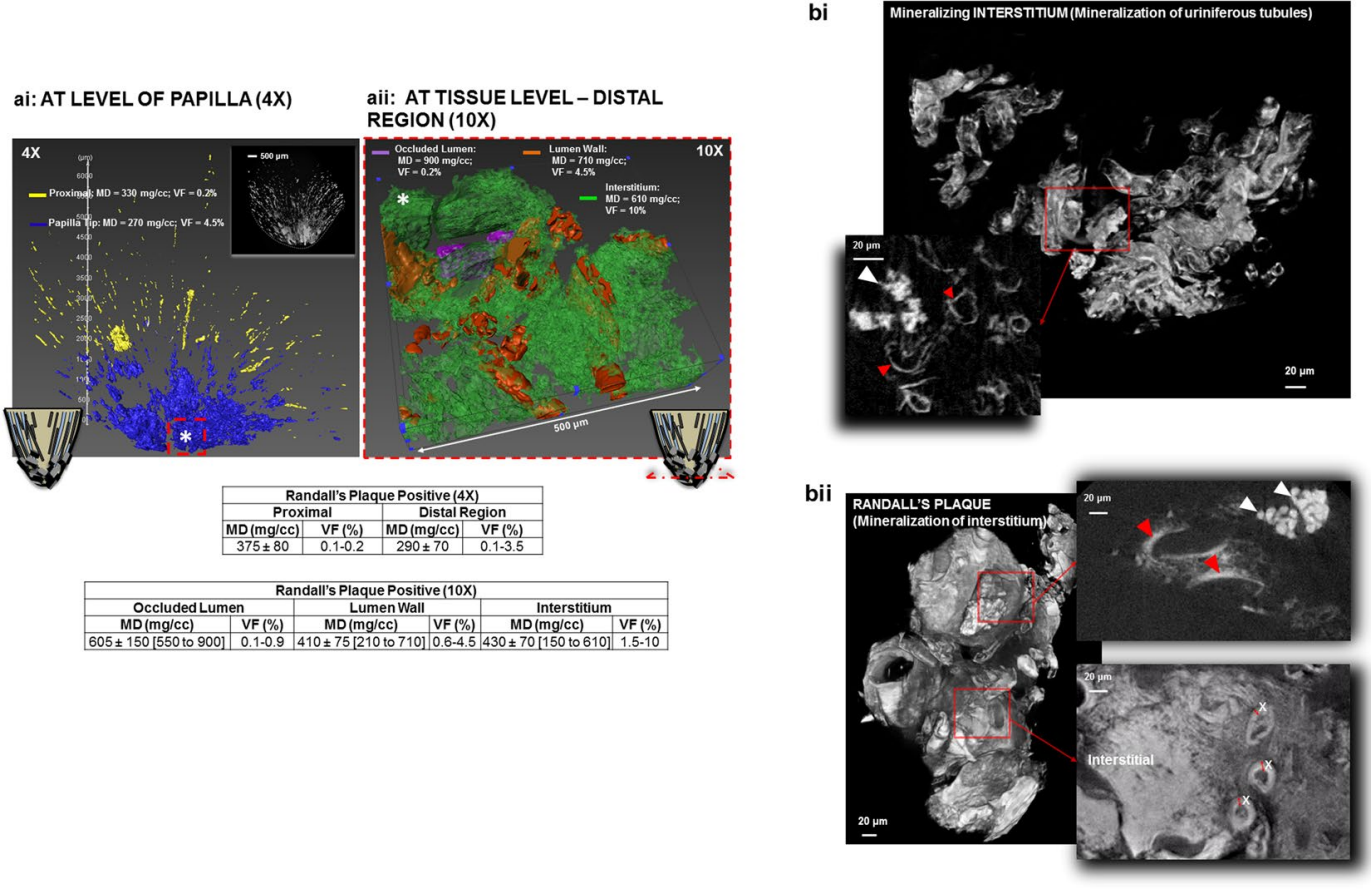
Volumes from micro-XCT with varying mineral densities and for different volume fractions are shown in (**a**,**b**). Schematic of the MPC is shown at the bottom of **ai** and **aii** with proximal occluded tubules (dark streaks) and interstitial mineralization in the distal region (mineralized blocks). Micro-XCT of representative MPC with proximal intratubular and distal interstitial biomineralization is shown as a inset in top right corner of ai. Segmentation of intratubular and interstitial minerals and respective mineral densities (MD, mg/cc) and volume fractions (VF) in the MPC (**ai**, 4X) and in distal region (**aii**, 10X) of the papilla are shown. Digital segmentation illustrates mineralized volumes in proximal (yellow) and distal regions (blue) of the papilla (**ai**). The red dotted box (*) represents the 3D subvolume used in **aii** with segmented mineralized volumes of occluded tubule (purple), tubule wall (orange), and interstitium (green). Average MD ± standard deviations [range] are shown in mg/cc, and VF values are the ratios of mineral volume to total volume expressed in percentages in the table below. (**b**) Distal interstitial mineralization at a higher magnification can be observed in the tubule walls (red arrow heads) of the loops of Henle in RP^−^ with minerals of higher densities (white arrow head) (**bi**), and in the interstitial matrix of RP^+^ with minerals of higher densities (white arrow heads), and in tubular walls (red arrow heads) and adjacent interstitium (bii).

**Figure 5:**
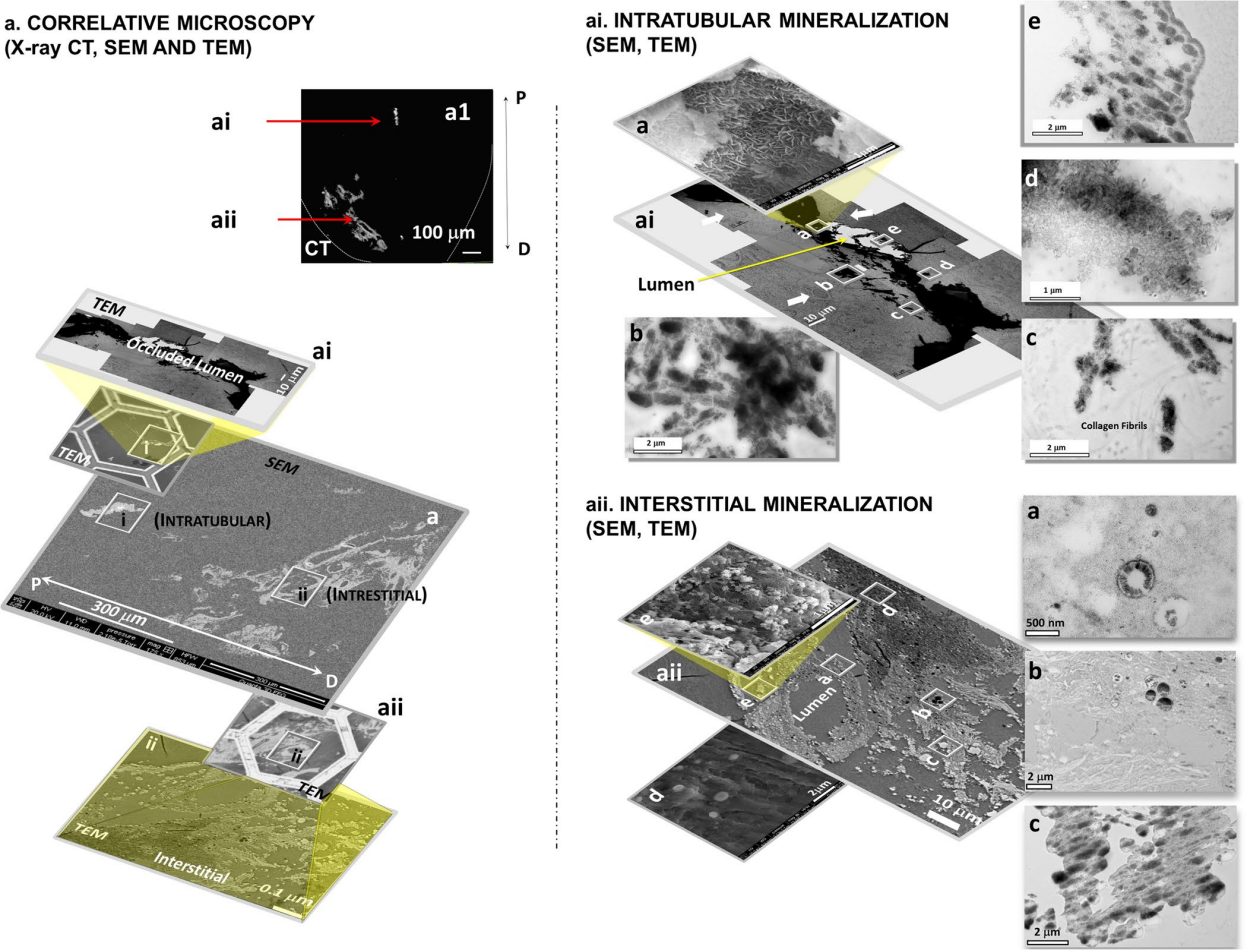
Contextual information on intratubular and interstitial biominerals is extracted by correlating data sets gathered from micro-XCT, scanning and transmission electron microscopy (SEM and TEM) techniques. Correlation between intratubular (**ai**) and interstitial (**aii**) biomineralization from data sets acquired with micro-XCT (**a**), and SEM and TEM (**ai**, **aii**) is performed. Regions **ai** and **aii** highlight intratubular and interstitial biominerals. Note regions a–e in **ai** and **aii** are subregions. In a1, region ai and subregions (**a**–**e**) in ai illustrate plate-like configurations of intratubular minerals. Note the association of minerals with collagen fibrils (**aiic**). In contrast, interstitial biominerals (**aii**) are spherical in nature whose cross sections illustrate concentric ring-like structures (**aii**, regions a–e). Spherical agglomerates noted (**aii**, regions a–e) also illustrate circular clusters in cross section (**aii**, regions a–c). D: Distal; P: Proximal.

**Figure 6:**
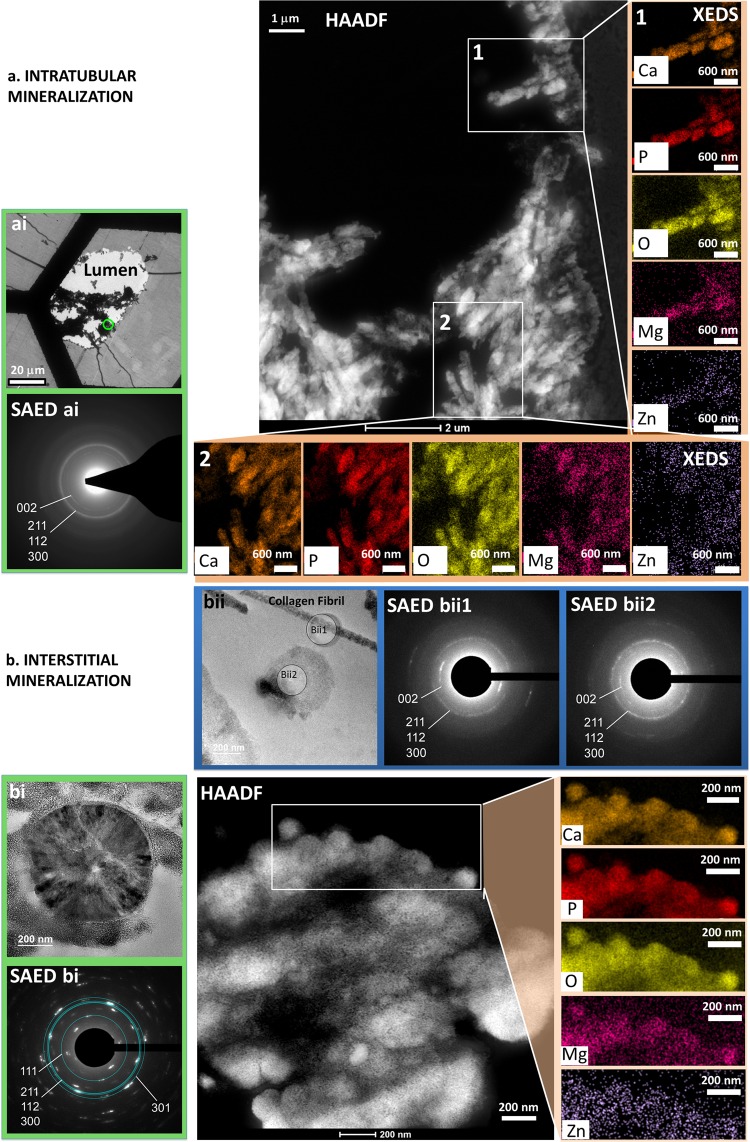
Structure and chemical composition of intratubular (**a**) and interstitial (**b**) minerals in the medullo-papillary complex (MPC). Images on the left (**a**) illustrate intratubular mineral in the proximal regions of the MPC at lower magnification (TEM, bright field) and via a selected area electron diffraction (SAED **a**) pattern (bottom) of the mineral aggregates outlined in green in (**a**). High-angle annular dark-field (HAADF) scanning TEM (STEM) illustrates a heterogeneous distribution of lower and higher atomic weight elements in regions 1 and 2. Elemental mapping by STEM X-ray energy dispersive spectrometry (XEDS) of regions labeled “1” and “2” in (HAADF **a**) identify calcium (Ca), phosphorus (P), oxygen (O), magnesium (Mg), and possibly zinc (Zn) (right column and bottom row). The results of quantitative analysis of the elemental maps are shown in Table S1. In contrast, interstitial minerals known as nanostones or calcified nanoparticles (CNPs) are imaged in distal regions of the MPC (TEM, bright field, bi) with the corresponding SAED pattern (SAED bi) also shown. Interstitial mineralization was identified with collagen fibrils (bii1) and nanostones (bii2). Respective SAED patterns show diffraction spots and indicate crystallites oriented along the collagen fibril (SAED bii1) and the presence of crystallites with no preferred orientation associated with nanostones (SAED bii2). A HAADF view of the interstitial minerals is shown in the large image below. Elemental mapping by XEDS of the highlighted region identified (Ca, P, O, Mg) and possibly Zn. Quantification results of elements in sub-regions marked 1 and 2 are shown in Table S1.

**Figure 7:**
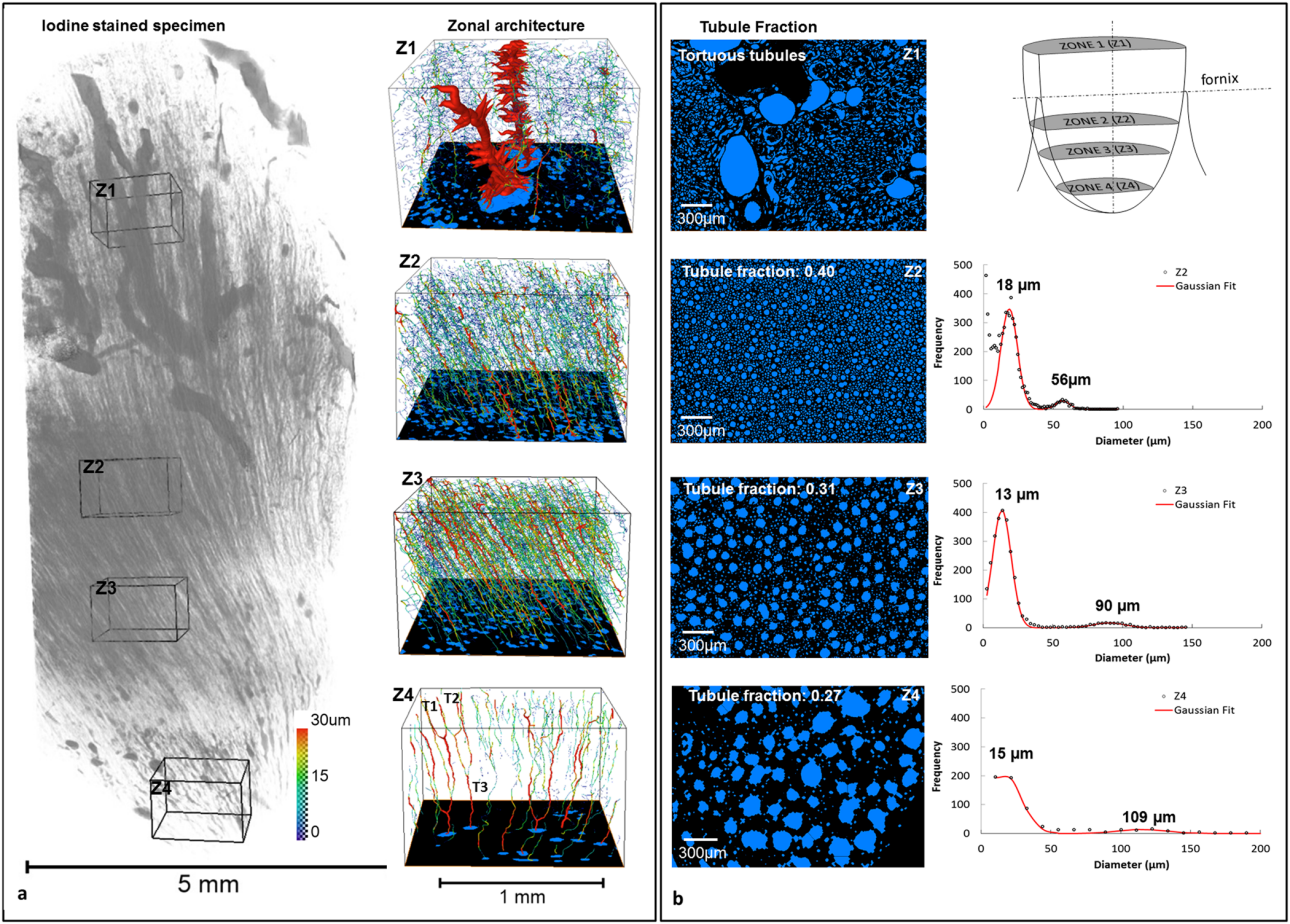
(**a**) Architecture of various zones of the human medullo-papillary complex. Inverse-tomogram of an iodine-stained specimen imaged using micro-XCT illustrated uriniferous tubules and large renal arteries/veins in the proximal portion of the MPC. Auto skeletonized images (right column) at four different regions (Z1, Z2, Z3, Z4 with a diameter range from 16–300 μm) after segmentation with virtual transverse slices along with diameter ranges are shown. Note that the tubules in Z4 are merged together successively providing insights into an increase in flow rate in the combined tubule (T_3_) compared to the flow rate of the merging tubules (i.e., T_1_, T_2_) where the diameter of the T_3_ is smaller than the sum of the diameter of T_1_ and T_2_. (**b**) Tubule fraction is defined as the volume of segmented tubules to the total volume of the tissue within each zone (zones 1 (Z1) through (Z4)). Note a shift in the peak indicative of detectable tubular diameter within respective zones.

Contextual information on intratubular and interstitial biominerals from excised renal MPCs at multiple length-scales is shown in Fig. 1 and as a Supplemental Video S2. Patient care X-ray CT at a magnification equivalent to macroscale lengths of meters to several millimeters illustrated the location of the kidneys (Fig. 1, Supplemental Video S2). The kidneys were digitally segmented to highlight the kidney stone, and further examination of the excised MPC from the kidney using higher resolution micro-XCT at 4X magnification detailed anatomically-specific mineralization (subvolumes 3 and 4 in Fig. 1c,d; subvolumes 1 and 2 in Fig. 2a,b). RP^−^ and RP^+^ MPC as seen by the micro-XCT are shown in Figs. 3 and 4. In all specimens, proximal mineralization was observed in RP^−^ specimens. At a higher magnification (10X) using micro-XCT, in subvolume 1, Fig. 2, intratubular minerals were observed as long streaks of “chalk”. In subvolume 2, Fig. 2, interstitium of the distal portion of the papilla illustrated circuitous macaroni-like mineral formations. These significantly different anatomically-specific mineralizing regions were consistently observed in all specimens including those that were thought not to contain RP when clinically examined endoscopically (Figs 2b and 3a). Proximally located intratubular minerals are shown in Figs 2 and 4ai. Distally located interstitial minerals are shown in Figs 3 and 4. Respective ultra/nanostructures of proximal and distal biominerals as seen by transmission and scanning electron microscopy techniques are detailed in Figs 5, S3a and S3b for intratubular, and Figs 5, S3c and S3d for interstitial minerals. Proximal regions of MPC in RP^−^ specimens consistently illustrated intratubular minerals (subvolume 1 in Fig. 2) in spite of the absence of RP. These minerals appeared to be plate-like particles (Figs S3a and S3b). Distal regions of the MPC in RP^+^ specimens contained mineralized interstitial matrix in addition to mineralized tubule walls, in spite of open un-occluded tubules (1–3 in Figs 3a and 5–7 in 3b, and regions in Figs S3c and 3d) illustrating clusters of minerals forming larger agglomerates.

### “Spectrum” of mineralization at multiple length-scales of MPC: mineral density variation between intratubular and interstitial minerals, and a spectrum of mineralization in the distal MPC as seen by a micro-XCT

Using X-rays and imaging at higher magnifications of 20X and 40X (Fig. 3a), RP^−^ revealed mineralized proximal tubules (both straight and convoluted). A “spectrum” of degrees of mineralization (changes in volume fraction of mineral and mineral density) within the distal region of RP^−^ specimens were observed (Figs 3 and 4) upon further examination of the distal regions of the MPC. At a lower magnification of 4X, proximal regions with occluded tubules had higher mineral density material (Fig. 4ai) compared to the interstitial distal matrix (Fig. 4aii). At a higher magnification of 10X, interstitial matrix illustrated various mineralized subvolumes (Fig. 4aii, red and white arrow heads in Fig. 4bi,bii) all of which were higher in their mineral densities compared to that measured at 4X magnification. This higher mineral density observed at 10X magnification within the tubule (white arrow heads, Fig. 4aii) also illustrated stone emerging from the mineralized interstitium (red arrow heads, Fig. 4bii) into the collecting system of the kidney (Fig. 4bii, white arrow heads along in the virtual sections taken from the same rendered volume). Markedly different mineral densities and volume fractions were observed in proximal versus distal regions of the MPC at lower and higher magnifications (Table in Fig. 4a). The interstitium, tubule wall and tubule were of different mineral densities and in any given specimen were of different volume fractions (Fig. 4a,b).

### Shape and type of anatomically-specific biominerals, and concentration of various elements within intratubular and interstitial biominerals

The location of respective site-specific minerals as observed on the ultrasectioned surface of the embedded specimen using an SEM (micrograph “a” in Fig. 5) was correlated to a virtual X-ray slice extracted from a reconstructed volume of the MPC (Fig. 5, regions 5ai and 5aii). Regions identified using SEM were further correlated with regions within the ultrasections on the TEM grids (regions i and ii in location a of Fig. 5 and subsequently regions 5ai and 5aii). TEM (Fig. 5, regions 5ai and 5aii) illustrated minerals within the lumen of tubules in the proximal region (Fig. 5ai) in contrast to mineral in the peri-tubular wall and interstitial matrix (Fig. 5aii), and minerals in tubule wall of mostly un-occluded tubules in the distal region of the MPC. The proximal minerals were spherical, but were made of plate-like radially oriented crystallites (region a in Fig. 5ai) in contrast to the distal interstitial spherical minerals which were of core-shell motifs (Figs S3a and S3b) as observed by both SEM and TEM. While SEM provided a 3D perspective of the topography of the crystallites (region a in Figs 5ai; S3a and S3b), TEM provided a visual of the cross sections of minerals dependent on the angle at which the specimen was sectioned (Fig. 5ai,aii). Regardless, the cross section illustrated the same geometry of minerals while the aspect ratio varied depending on the sectioning angle and the location and orientation at which mineral was identified. Minerals were interspersed with degraded collagen fragments (region c in Fig. 5ai). In the proximal region (Fig. 5ai), cellular matter appeared to remain intact (white block arrows in Fig. 5ai), while the lumen exclusively was filled with minerals (black region in Fig. 5ai). In contrast, in the distal regions, concentrically layered mineralized nanospherulites were associated with collagen fibrils (regions d and e in Fig. 5aii) of the interstitium, and within the tubule wall. Clusters of nanospherulites were observed (Fig. S3c and d as seen by SEM, and Fig. S4 as seen by TEM), and their geometry was confirmed and correlated by visualizing their cross sections as determined by the TEM and SEM (Figs 5, S3,S4). Note that the diagonal striations in region c in Fig. 5aii are specimen preparation artifacts such as “chatter” from the ultramicrotome.

Crystallites identified by TEM were analyzed in more detail using selected area electron diffraction (SAED) and scanning TEM (STEM)-based elemental mapping by X-ray energy dispersive spectrometry (XEDS) (Fig. 6). SAED patterns of the intratubular crystallites revealed diffuse concentric rings indexing to the 002, 211, 112, 300 lattice planes of apatite (Fig. 6a). STEM-XEDS of the intratubular mineral mapped calcium, phosphorus and oxygen to the crystallites in relative amounts that was consistent with the stoichiometry of hydroxyapatite (Table S1). Trace amounts of magnesium and zinc also were detected, albeit the level of zinc was close to background. SAED patterns for interstitial mineralization in regions bi and bii in Fig. 6b revealed discrete diffraction spots for nanoclusters (bi) and preferentially oriented mineral along the collagen fibrils (bii1 in region bii). Nanoclusters with darker radially dominating bands (Fig. 6b, region bi) indicated polycrystalline material with no texture, compared to those observed in region bii as shown in figure 6b, where textured pattern owing to 002 preferential orientation was observed. The 211, 112 and 300 lattice reflections indexing to apatite are also observed in the interstitial nanospherulitic structure (Fig. 6b, region bi). As in the intratubular case, quantitative analysis of the elemental maps of interstitial biominerals closely matched the stoichiometry of hydroxyapatite with an atomic ratio of Ca/P close to 1.67 suggesting a typical apatite structure regardless of the shape of the aggregate (plate-like or nanospherulitic structures) (Table S1). The leading mineralizing fronts of clusters identified in the interstitial regions appeared to contain trace amounts of magnesium and zinc (albeit close to background levels of zinc) whereas the “bulk” interstitial mineral with varying levels of crystallinity identified by calcium to phosphorus ratios (Table S1) next to the clusters did not (Table S1b regions 1 and 2).

### Functional zonation: Tubular network within a human MPC as seen by a micro-XCT

Zones of varying tubule diameter approximately 10–30 µm were identified from digitally reconstructed volumes of the MPC. Figure 7a illustrates the network of tubules of varying diameters, and Fig. 7b illustrates two distinct peaks for tubule diameters at each zone respectively. Peaks depicting lower diameters overlapped with those that were measured using iodinated specimen, while the second peak at each zone depicting larger diameter was more indicative of collecting tubules and ducts in the MPC, and as evidenced by anatomically-specific zonation.

## Discussion

The motivation for this study was guided by the unique intrinsic architecture of the MPC. Architecture-guided fluid flow suggests that upstream biomineralization events can lead to downstream commonly observed larger mineral aggregates at the distal regions of the human renal papilla and ultimately develop into urinary stones. Traditional dogmatic variants of these site-specific biominerals include nephrocalcinosis (biominerals in the renal parenchyma) distinct from nephrolithiasis (urinary stones in the renal collecting system). Combining digital spaces obtained from patient care X-ray computed tomography to benchtop micro-XCT (Fig. 1) facilitated correlation of data at respective length scales and allowed visualization of biominerals at site-specific anatomical locations within larger 3D landscapes of kidneys. Application of materials science approaches combined with higher resolution imaging using X-rays, electron, and light microscopy techniques revealed critical insights into pathophysiological processes underlying nephrocalcinosis and nephrolithiasis, and a template for targeted therapy approaches. Many factors including gene-products stimulated by bioenergetics and environmental factors regulate biomineralization of the renal papilla^16^. Our focus however will predominantly be on the results obtained through multiscale mapping of biominerals within several human MPC excised at singular time points.

The ~1 cm long MPC is nature’s bioengineered functional unit of a ~12 cm long macroscale kidney. At any given transverse section, a multitude of urinary (nephrons) and vascular (vasa recta) tubular networks with diameters ranging from 15 through 300 μm form a forest of tubular networks. The core feature of the renal papilla is the intricate network between a forest of 15–20 μm smaller diameter shorter and longer nephrons (twigs), and the vasa recta that skirt around these nephrons^1,17–19^. The proximally located shorter length nephrons^1,2,20–26^ are peripheral based on the paraboloid-like shape of the renal papilla, while longer length nephrons are more centrally located and run deeper into the distal regions of the MPC tip. This nephron-vasa rectae network merge into 30–60 μm larger diameter tubes (branches) ultimately connecting to a 100–250 μm larger diameter collecting ducts (tree trunk) (Fig. 7). Within this forest of tubules of MPC, several functional units that aid in ion exchange and urine concentration can be identified; (a) a urothelium in association with a vasa recta forming a primary unit; and (**b**) several urothelial tubules with several vasa recta forming a larger secondary cluster/functional unit^17–29^ within respective zones of the MPC. This primary to higher ordered tubule clusters is one of nature’s conserved signatures, and forms a physical continuum for fluid flow in the MPC through various branching yet interconnecting networks, through a variety of cell-cell and cell-matrix interactions^30^. Based on paraboloid-like MPC, fundamental principles of fluid dynamics dictate significantly lower flow velocity at the periphery of the MPC. With lower fluid velocity at the periphery of the parabolic MPC in combination with lower medullary blood flow rates needed for concentrating urine^30^, an earlier occurrence of proximal mineralization compared to the centrally located tubules is conceivable. Additionally, based on “functional zonation” of the MPC^1,17–19^, the higher volume ratio of urinary (nephrons) and vascular tubules to the peritubular interstitial matrix proximally (Fig. 7), increases the propensity for proximal intratubular mineralization compared to a lower ratio of tubules to matrix in the distal regions of the MPC (Fig. 7).

Interestingly, within this architecture-guided continuous flow, there appears to be a transition zone free of detectable minerals (Figs 2 and 4), and this occurs between the mineralizing proximal and distal volumes of the MPC. It is postulated that at this interface the well documented changes in nephron tubular diameter (i.e. large to small, and then small to large diameters along with U-bends in loops of Henle) result in altered tubular pressure over the length of the nephron. This change in pressure due to change in diameter over the length of the nephron is converted to a significant increase in kinetic energy of the fluid. From an engineering perspective this effect is known as the Venturi effect^31^. The Venturi effect can result in a transition from relatively steady state flow to a dynamic urine flow and could be nature’s unique fluid propulsion of urine in the MPC (Fig. 9, A). Consequently changes in local pressure in addition to ionic concentration and pressure gradients over the length of the tubules (radial and longitudinal chemical and pressure gradients – ~320 mOsm in the interstitium of the cortex to ~1200 mOsm at the tip; and ~120 mm Hg at the cortex and ~5 mm Hg at the tip of the papilla) permit flow through the forest of tubules through various functional zones of the MPC (Fig. 7)^2,17,18,32^. Over time, shifts in chemical gradients both radially and longitudinally could regulate pressure gradients along the length of the MPC.

Locally, biomineralization within the MPC could be induced by the principles of physical chemistry and cells^16^. It continues to be a challenge to investigate the influence of architecture-guided flow dynamics on cell behavior and various types of cells, and vice versa in the space continuum of the MPC. The physical chemistry based mineral formations could be influenced by architecture and can be explained within the context of results gathered by using a correlative microscopy approach. It was interesting to note that the shapes of minerals were anatomy-specific, in that the proximal biominerals were different in their configuration compared to distally located biominerals. Although biominerals were identified proximally, results do not provide the cause for intratubular biomineralization. Based on observed proximal intratubular and distal interstitial biominerals, what are the nidi or prenucleators that lower the activation energy for biomineralization to occur?

Observed results are culminated effects of multitude of factors, the observed end-stage event as seen by a microscope could be a result of anatomy-specific biological and physical chemical processes. Cell-based autoregulation of intracellular and extracellular ions/proteins and local pH can also regulate type and rate of biomineralization^30,33,34^. Blood filtration through the glomerular apparatus, and subsequently post glomerular modification including urine concentration through theorized counter-current exchange of anions (e.g. Cl^−^, SO_4_^2−^, HPO_4_^2−^ HCO_3_^2−^), cations (e.g. Na^+^, Ca^2+^, K^+^, NH_4_^+^, Mg^2+^), and water is a fundamental process that is regulated by virtue of crosstalk between zone-specific cells. Under normal conditions, the proximal tubules are also lined with ruffle-ended epithelial cells which mediate pH and prevent biomineral aggregation. The concentration, mineral density variation, and degree of crystallinity of intratubular minerals is related to the overburdened epithelial cells (with carbonic anhydrase) prompting shifts in pH and changes in the ratio of carbonate to phosphate (resulting from epithelial cell generated bicarbonate - HCO_3_^2−^, and local serum phosphate - HPO_4_^2−^). This argument is further strengthened by *in vitro* based experiments with increasing concentration of calcium carbonate where spherulitic calcium phosphate nanoparticles with plate-like configuration as seen in the cross section (Figs 5 and 6, S3a,S3b) also were observed^35^.

The observation of intact cells was limited to the proximal region of the renal papilla in contrast with the distal aspect where no intact cells in the interstitium were observed, and markedly increased collagen fragments were seen. Plate-like calcium phosphate intratubular minerals that also were observed in addition to spherical minerals (Figs 5 and 6, S3a,S3b) were of amorphous (observed but not shown) and crystalline types (Fig. 6) with low concentrations of magnesium and zinc (zinc signal close to background) (Fig. 6, Table S1). In contrast, the minerals in the distal interstitial matrix were distinctly crystalline in nature, and were of spherical morphology with concentric circular configuration in cross section, that is, core-shell ultrastructure. While we cannot rule out the presence of oxalates and carbonates, we can conclude that the majority of intratubular and interstitial crystallites examined in the MPC are phosphate-based albeit a plausible calcium carbonate precursor. However, the relative amounts of calcium carbonate to calcium phosphate in the proximal and distal locations of the MPC are yet to be determined.

Distal interstitial calcification was always identified together with proximal/peripheral intratubular biominerals^36^ and never by itself. Additionally, as reported earlier, distal biominerals were found to be spherulitic^36^ and could be associated with the lymphatic system^17–19,37^ as it maintains water balance and hydrostatic pressure within the MPC; distally located biominerals were similar to those observed in the lymphatic system of a lung^17–19,38–41^. We contend that there lie lipid-laden interstitial cells, and pericytes that are stimulated due to shifts in pressure gradients. Mechanobiological activity of lipid-laden interstitial cells and pericytes can collectively promote lipid-protein based mineralization. Additionally, lipid-laden interstitial cells at distal zones of the MPC as identified in rats also contain lipid droplets, and contact and/or connect loops of Henle and vasa recta as they increase in number toward the tip of the papilla^37^. This corroborates with the observation of concentrically layered calcified nanoparticles with specific crystal morphology and crystallographic orientation along the long-axis of the collagen fibril (Fig. 6); the presence of lipid-laden cells in the human distal renal papillary tip should be investigated. Additionally, vesicle mediated mineralization also can be used to explain the concentric spherical shape of calcified nanoparticles^42^. The layered structure of the calcified nanoparticle (Figs. 5 and 6, S3c, S3d, S5) suggests an alternative and repetitive and organized process during mineralization, and could be more of a lipid-protein collective effort. The observed higher ordered microscale mineral apatite aggregates that were predominantly crystalline could be assemblies of nanometer vesicles (Fig. 6) or can be attributed to Ostwald’s ripening (dissolving of the smaller more soluble crystals and re-deposition of the dissolved material on the growing faces of the larger, less soluble crystals)^43^. Regardless of the biomineralization process, precise localization of the organics within the stratified layers that form calcified nanoparticles (CNPs) will provide insightful information, specifically in deriving a targeted approach through pharmacological interventions to prevent aggregation of higher ordered calcified bodies.

It should be noted that there is predominance of certain types of motifs in the proximal and distal papillary regions based on higher resolution correlative imaging. Proximally and distally located minerals are predominantly spherical in nature. However, the proximally located higher ordered spherical structures resulted from the radial arrangement of crystallites. In contrast, the distally higher ordered spherical structures were commonly from the core-shell type arrangements of crystallites (Fig. 5). The predominance of these higher ordered structures with varying arrangement of crystallites in the proximal and distal locations of the renal papilla does not exclude mineral nodules with other arrangements of crystallites, and these structures are shown in Supplemental Figures (Fig. S5).

The association between morphologically distinct proximal to distal biominerals has been proposed to occur as a result of mobilization of the proximally forming particulates into the distal regions of the papilla according to the Anderson-Carr-Randall progressive biomineralization theory^36^. Based on results in this study, we propose an alternative theory of a mechanobiological link between proximal intratubular and distal interstitial biominerals in the MPC.

**Figure 8:**
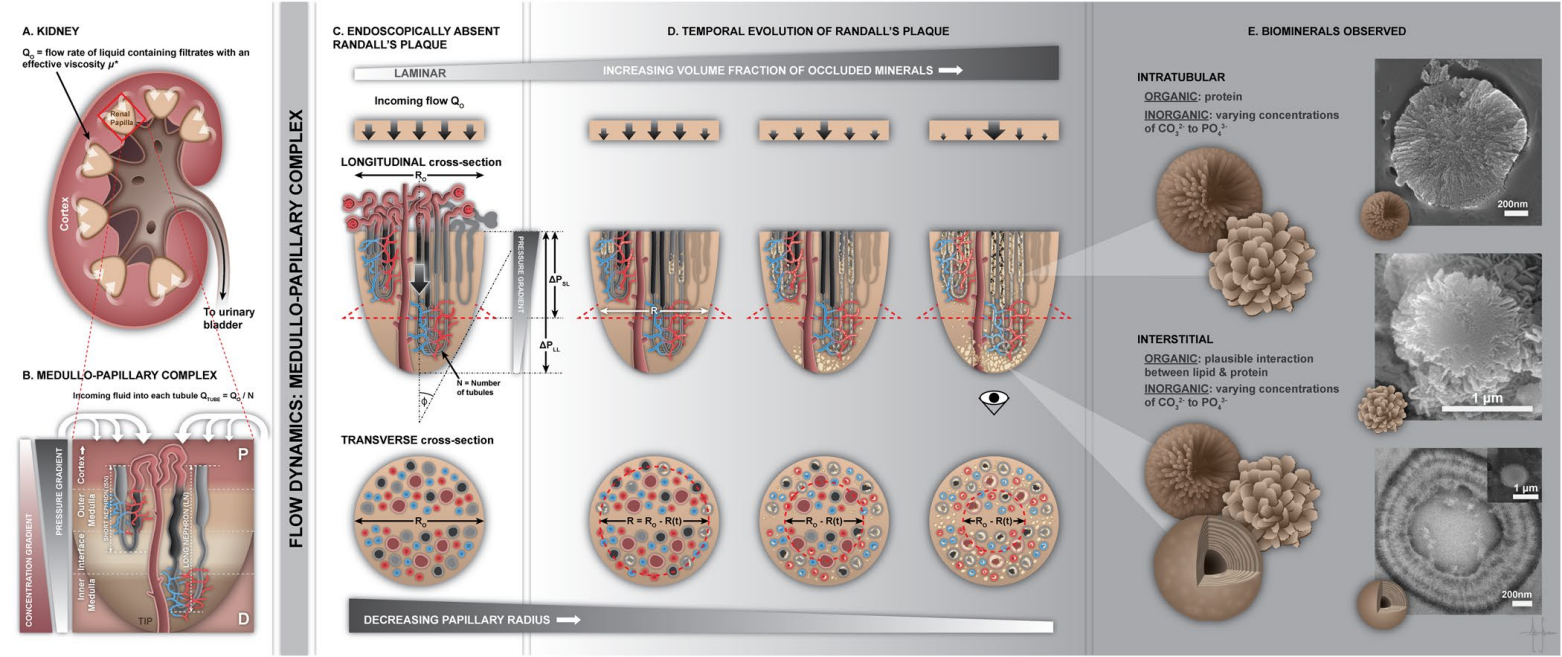
Architecture-guided bioengineering model to explain intratubular renal biomineralization. I. Proximal intratubular mineralization of the medullo-papillary complex (MPC) (**A**). Schematic of a kidney describes flow characteristics in a paraboloid MPC (radius R_h_ = R_o_ − h*tan(θ); θ is the angle of the complex). Q_o_ is the incoming fluid flow rate into the MPC and flows into the many short looped (SL) peripherally located (cortical nephron, CN), and the long looped (LL) centrally located (juxtamedullary nephron, JN) tubules; collective number N (**C**). Existing ‘loads’ along the length of the MPC include pressure and chemical gradients (**B**). Fluid flow at the periphery of the paraboloid MPC is lower than the flow in the centrally located tubules. Over time, filtrates accumulate and result in a decrease in functional radius (‘R = R_h_ − R(t)’) (**D**).

**Figure 9:**
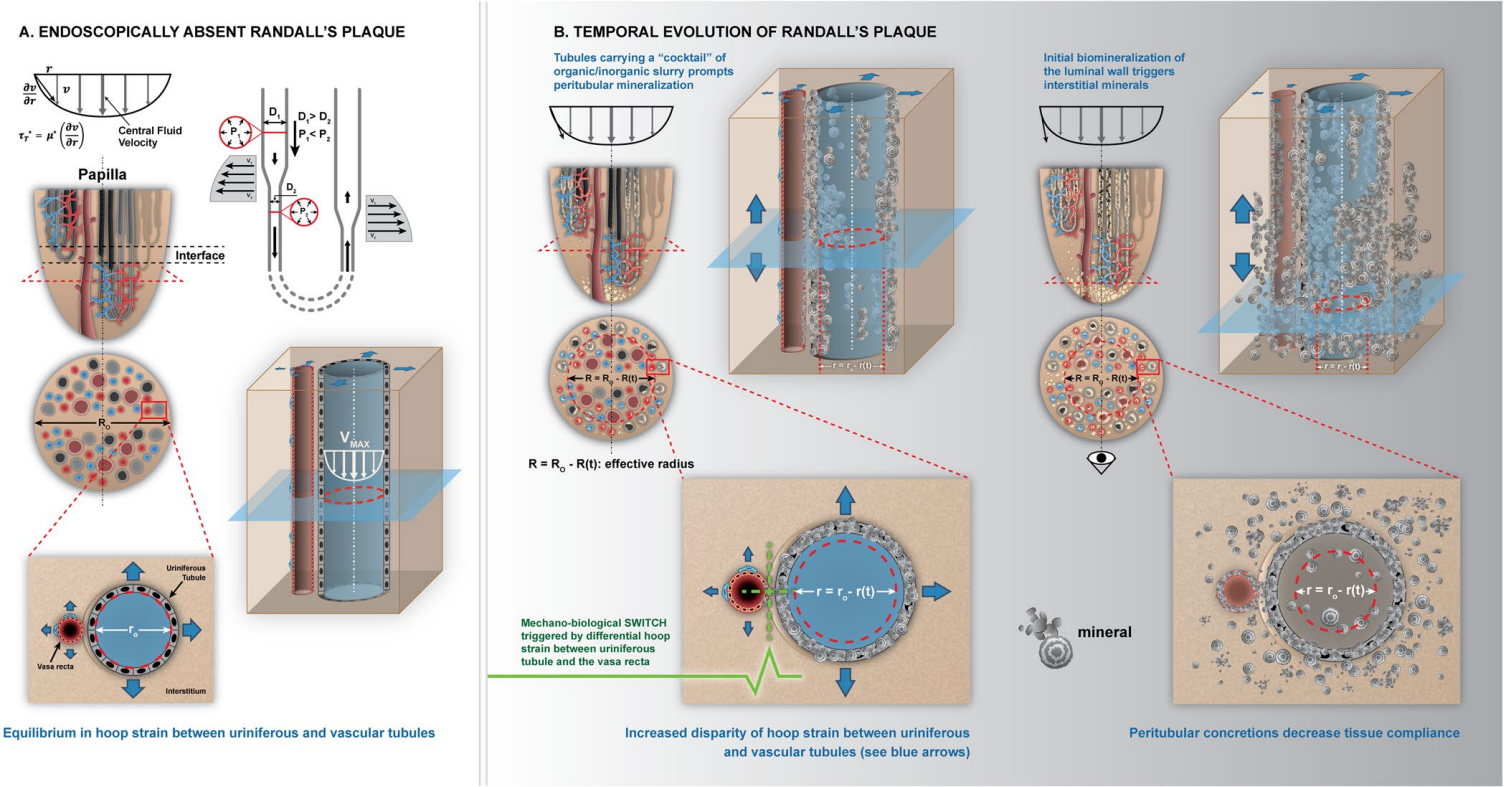
Architecture-guided bioengineering model to explain interstitial renal biomineralization. Distal interstitial mineralization of the medullo-papillary complex (MPC). (**A**) MPC (no Randall’s plaque) with uriniferous tubules and vasa recta, along with velocity profiles (top) and transverse cross section with functional radius (R_o_) are shown. The flow velocity profile within the uriniferous tubule with radius ‘r_o_’ (blue) and vasa recta (pink) is indicated by minimum velocity near the wall and maximum central flow velocity with minimal to no accumulation or tubular obstruction. The resulting shear stress in the tube with certain radius is a function of fluid viscosity “ν”, and a differential in velocity. The decrease in functional radius of the MPC due to occlusion of peripheral tubules (Fig. 8) results in subsequent local biomineralization events (**B**). The differential in circumferential strains resulting from progression of peritubular biomineralization within both uriniferous tubules and juxtaposed vasa recta trigger a mechano-biological switch. This switch initiates the progression of peritubular to interstitial biomineralization through expression of osteogenic markers by differentiating cells in these local environments. These events decrease tissue compliance that can further decrease the functional radius of the MPC.

From a bioengineering perspective the schematic Figs 8 and 9 illustrate existing systemic ‘loads’ or burden on short and long nephrons (SN, LN) which also include pressure (120–50 mm Hg) and chemical (300–1200 milli-osmols) (Fig. 8, B) gradients from the cortex to the tip of the papilla^32^. As a consequence of the paraboloid architecture of the papilla, peripherally located nephrons are shorter compared to centrally located longer nephrons^32^. As a result, the rate of filtrate accumulation in the shorter tubules is higher than in the longer but centrally located tubules (Fig. 8, C). This filtrate accumulation is compounded by relatively steady state flow in the proximal descending tubules compared to a plausible dynamic flow in the thin loops of Henle. The temporal evolution of the change in MPC function (Fig. 8, D) can be approximated based on the form-function relationship and by using the principles of fluids dynamics. The functional radius “R” of the MPC over time “t” is approximated as ‘R = R_o_ − R(t)’ where R_o_ is the initial functional radius of the MPC (Fig. 8, D).

The schematic Fig. 9 illustrates a normal tubule with laminar flow (velocity, v) of fluid with viscosity µ* and with minimal to no accumulation or tubular obstruction (Fig. 9, A). Accumulation of filtrates on the wall of the tubule with radius ‘r’ occur due to the decreased velocity along the walls, and that flow in the proximal and peripherally located tubules is preferentially lower compared to centrally located tubules. As particulates accumulate, the functional diameter of the papilla is reduced. This will result in an aberration in flow and will alter the overall pressure gradient and in turn the pressure specifically at the interface between the proximal intratubular and the distal interstitial biominerals (with higher density of Venturi elements resulting from descending thick “D1” to thin “D2”, and ascending thin to thick). This implies that perturbations at the level of the papilla prompts changes in flow dynamics within the tubules and tubular pressures and the juxtaposed vasa recta which is illustrated in Fig. 9.

The effective change in radius of the papilla owing to peripheral tubule occlusion due to accumulation of filtrates in a fluid of viscosity ‘µ’ and flow rate ‘Q’ can fundamentally be modeled by Poisueille’s equation which is governed by the fourth power of radius ‘R’ of the papilla in the denominator and length ‘L’ of the tubule in the numerator ${\rm{\Delta }}P\propto \,\frac{\mu LQ}{{R}^{4}}$ . For a small change in R, a large shift in pressure gradient ‘Δ*P*’ can occur in the MPC. The change in pressure is experienced by the cells within regions between the nephron and the vasa rectae. The distensibility of these tubules changes as minerals accumulate in the tubules. This change propagates outside of the tubule into the adjacent vasa recta and subsequently over time into the surrounding interstitium (Fig. 9, B). It is here that we postulate a mechanobiological switch where the differential in circumferential/hoop strain prompted due to changes in flow dynamics and wall characteristics of the uriniferous tubule will in turn alter the distensibility of the vasa recta. The differential in circumferential strain between the small diameter pressurized vasa recta and the mineralizing nephrons is identified as the switch (Fig. 9, B) would turn on pericyte and/or a smooth muscle cell into osteoblast-like cell or produce osteogenic markers rendering the environment conducive for mineralization. However, mineralization of the plaque can happen both inside and outside the tube at the distal tip of the papilla. While the pressure and concentration gradients at the level of the papilla underline the biomechanics on the renal papilla, the shifts in cell-regulation identified by the shifts in these gradients can be better understood through principles of mechanobiology. We postulate that from a mechanobiology perspective, the interface between the proximal intratubular and the distal interstitial biominerals that originally augmented propulsion over time will ultimately mineralize.

Proximal intratubular biominerals were distinct from distal interstitial biominerals, and were spatially visualized by virtue of a mineral-free interface between proximal and distal extracellular matrices (Figs 1–4 and 7). At this interface, Venturi-like elements resulting from varied and changing tubule diameters exist and promote mineral-free zone; a change from steady state to dynamic fluid flow conditions. However, over time this mineral-free interface also could be impaired after mineralization occurs in a certain volume fraction of proximal tubules and distal interstitial matrix. Owing to the mineralization of the luminal wall, distal mineralization is not a result of flow of proximal particulates forming larger agglomerates in the distal regions of the MPC as indicated by Anderson-Carr-Randall progression theory. Given that distal interstitial minerals were observed only in the presence of proximal intratubular biominerals, it is postulated that the architecture of the MPC (Figs 2–7) could link proximally distinct intratubular occlusion with distal interstitial mineralization via a mechanobiological switch located at the mid papillary interface. The mechanobiological switch can in turn stimulate distally located pericytes and other cells that can transdifferentiate into bone-like cells or express osteogenic matrix molecules resulting in distal interstitial mineralization (Figs. 8 and 9). Over time, the distal interstitial mineralization can emerge into the calyx, thus enabling the link between nephrocalcinosis (intratubular) and nephrolithiasis (kidney stone). However, differences in anatomically-specific mineral structures plausibly owing to differences in calcium carbonate and calcium phosphate concentrations, and influence of organics including lipids will likely require different forms of targeted interventions.

## Materials and Methods

MPCs were excised from kidneys of patients undergoing nephrectomy following a protocol approved by the UCSF Committee on Human Research Protection Program, IRB # 14–14533, and were selected from a larger set of specimens from patients (N = 20) following *ex vivo* scanning using high resolution micro-XCT (Micro XCT-200, Carl Zeiss Microscopy, Pleasanton, CA). All patients provided written informed consent for the collection of specimens to be used for research purposes. No identifiable images were taken and all elements of the research protocol, relevant guidelines, and regulations were strictly adhered to during the course of the study. MPCs from kidneys with a history of chronic stone formation, chronic infection, hydronephrosis/obstruction, and/or cystic kidney disease were excluded. All specimens were examined for Randall’s plaque using a stereo light microscope and were categorized as RP^+^ with grossly visible plaque compared to those that did not illustrate Randall’s plaque as RP^−^.

### Specimen preparation, high resolution micro-X-ray computed tomography

The workflow of specimens is illustrated in Figure S1. Specimens were fixed overnight in 10% neutral buffered formalin (NBF, Richard-Allan Scientific, Kalamazoo, MI), washed twice in phosphate-buffered saline (PBS), then dehydrated with graded ethanol solutions prepared from dilution in Milli-Q purified water (50% to 100%) followed by imaging using micro-XCT at various magnifications of 2X (10 µm/voxel), 10X (5 µm/voxel), 20X (1 µm/voxel), and 40X (0.5 µm/voxel) with 1200 slices per each volumetric reconstruction. The digitally reconstructed micro-XCT volumetric data were further analyzed using AVIZO software (9.0.1, FEI, Hillsboro, OR).

To image the architecture of the human MPC, of the 20, one of the specimens was stained with iodine. It is known that vasculature can be revealed by iodine^44^. The MPC was carefully isolated from a fresh nephrectomy specimen, fixed overnight in 10% neutral buffered formalin (NBF, Richard-Allan Scientific, Kalamazoo, MI), washed three times in 1X phosphate-buffered saline (PBS), and was scanned using micro-XCT after staining with 1.5% iodine (Alfa Aesar, Ward Hill, MA) for 2 hours. After washing with ethanol three times, the stained papilla was scanned using micro-XCT.

### Transmission electron and scanning electron microscopy techniques

Following XCT-scanning, specimens were subjected to infiltration of LR-white resin (Electron Microscopy Sciences, Hatfield, PA). The infiltrated specimen was kept in a gelatin capsule (Electron Microscopy Sciences, Hatfield, PA) and polymerized for 2 days at 60 °C. 80 nm thick sections were cut using a diamond knife and an ultramicrotome (Reichert Ultracut E, Leica Microsystems, Inc., Buffalo Grove, IL)^45^. Specimens were collected on formvar/carbon coated Ni grids and were examined using transmission electron microscopes (Libra 200, Carl Zeiss Microscopy and TitanX, FEI) at an accelerating voltage of 200 keV (Libra) and 80 keV (TitanX). The TitanX is additionally equipped with an FEI Super-X quadrature detector (solid angle 0.7 sr) for X-ray energy-dispersive spectrometry. Amorphous and crystalline states of minerals within volumes of proximal and distal regions of the MPC were examined using bright-field transmission electron microscopy (TEM) and selected area electron diffraction (SAED) (Libra), and by high-angle annular dark-field (HAADF) scanning TEM (STEM) combined with elemental mapping using STEM-based X-ray energy dispersive spectrometry (XEDS) (TitanX). The X-ray detector of the TitanX was calibrated using a copper standard and the XEDS data analyzed using Bruker Esprit software (Bruker Nano Analytics). The sectioned surfaces of the resin embedded specimen blocks also were imaged under the light microscope (BX51, Olympus America, Inc.). The locations of TEM sections within volumes of the MPCs were identified by correlating light micrographs of sectioned surfaces with virtual sections in X-ray tomograms collected from the same specimens prior to LR-white resin embedding (Supplemental Fig. S1). The ultrasectioned block surface also was examined with a scanning electron microscope (SEM) (Sigma VP500, Carl Zeiss Microscopy, and Quanta 3D FEG, FEI) at 5 to 20 keV.

### Statistics for Mineral Density and Tubule Diameter

#### Mineral Density

Variation in mineral density was assessed through segmentation with the post-processing AVIZO software (9.0.1, FEI, Hillsboro, Oregon) using water-shed algorithm and via a histogram based on intensity differences within the proximal and distal regions of the MPC. The water-shed method utilizes an iterative computational process to segment regions of distinct composition based upon Hounsfield units (HU). In short, a ‘seed’ region is manually selected, and the computer segments the specimen through comparison of neighboring voxels by their HU. When sufficient difference in HU are identified (based upon user specifications and comparison to the mean HU value of the region) the computer will delineate boundaries within the specimen. Gaussian fits were used to identify tubular structures as described in methods section 2.

#### Tubule Diameter

Customized MATLAB (R2013b, MathWorks Inc., Natick, MA) codes were used to analyze diameter of tubules by using histology sections taken at various functional zones of the MPC including the renal tip. A histogram of the tubule diameters showed several peaks and Gaussian functions were generated to best fit the distribution with the peak as a representative value for tubule diameter. Significant differences in peak values were considered if the absolute difference between peak values was at least the sum of two half-widths at the half-maximum of the peaks.

## Electronic supplementary material

Figure S2

Supplemental Information
