# Supplementary material for: Architecture-Guided Fluid Flow Directs Renal Biomineralization
Source: Sci Rep. 2018 Sep 21;8:14157. doi: 10.1038/s41598-018-30717-x (PMC6155006; doi:10.1038/s41598-018-30717-x)
Supplement: Supplementary file 2 — Supplemental Information [file 41598_2018_30717_MOESM2_ESM.docx]

# Architecture-Guided Fluid Flow Directs Renal Biomineralization

Sunita P. Ho^*,1,5^ PhD, Ling Chen^1^ PhD, Frances I. Allen^2,3^ PhD, Ryan S. Hsi^4^ MD, Alex R. Shimotake^1^, Scott V. Wiener^5^ MD, Misun Kang^1^ PhD, Andrew M. Minor^2,3^ PhD, Marshall L. Stoller^5^ MD

^1^Division of Biomaterials and Bioengineering, School of Dentistry, University of California San Francisco, CA 94143; ^2^Department of Materials Science and Engineering, University of California Berkeley, Berkeley, CA 94720; ^3^National Center for Electron Microscopy, Molecular Foundry, Lawrence Berkeley National Laboratory, Berkeley, CA 94720; ^4^Department of Urologic Surgery, School of Medicine, Vanderbilt University, Nashville, TN 37232; ^5^Department of Urology, School of Medicine, University of California San Francisco, CA 94143

***Materials and Corresponding Author:**

Sunita P Ho, Ph.D.

Department of Urology

School of Medicine

Department of Preventive and Restorative Dental Sciences

School of Dentistry

University of California San Francisco

**Mailing Address:**

707 Parnassus Avenue, D 3212

University of California, San Francisco

San Francisco, CA 94143

**Lab Address:**

Health Sciences West (HSW) 813

513 Parnassus Avenue

University of California, San Francisco

San Francisco, CA 94143

[sunita.ho@ucsf.edu](mailto:sunita.ho@ucsf.edu)

Phone: 415-514-2818

Fax: 415-476-0858

**Funding Support**: NIDCR/R01DE022032 (to SPH), R21 DK109912 (SPH, MLS), and NIH: NIDDK/P20DK100863 (to MLS); Departments of Preventive and Restorative Dental Sciences, School of Dentistry; Department of Urology, School of Medicine, UCSF

**Running Headline**: Architecture-Guided Renal Biomineralization

**SUPPLEMENTAL MATERIAL**

- - - 1. **MATERIALS AND METHODS**
  1. **Specimens used in this study**

All participants provided both verbal and written consents to participate in this study. Institutional approval for both the study itself and the manner in which consent was obtained from Human Research Protection Program (HRPP) at the University of California, San Francisco, was recorded under institutional review board (IRB) approval number 14-14533. Participants discussed the details of the study at their clinical visit where questions were answered by research team members and their verbal and written consents to participate were obtained. All study consents were then scanned and recorded into our data warehouse and the paper copies were destroyed in a secure fashion in accordance with institutional standards. Papillary specimens (n=14) were obtained from patients undergoing nephrectomy for the diagnosis of renal mass, presumed to be renal cell carcinoma. Patients who were thought to have transitional cell carcinoma were excluded from this study. During harvest of the papillae, an anatomically normal portion of the kidney, distant from the renal mass, was selected. In accordance with current dogma, renal tissue distant from renal cell carcinoma is generally thought to be normal unless subject to mass effect from the tumor. Typically, if a lesion is small enough and it is possible to reconstruct the kidney after mass excision, partial nephrectomy is preferred according to AUA guidelines^1^. This is based upon a multitude of published literature suggesting that not only will renal tissue that is preserved continue to function at a near normal level, but that oncological outcomes are excellent in terms of local recurrence ^2,3^. Unfortunately, in this patient cohort, radical nephrectomy was indicated based upon tumor anatomy (for instance the tumor was too close to the blood vessels to allow safe excision, or reconstruction was not possible) and salvage of the kidney was not possible^4^. Neo-adjuvant chemotherapy was not employed in any patient before nephrectomy in accordance with standard of care. Excised tissue distant to the tumor was examined at nephrectomy, had a grossly normal appearance, and was felt to be appropriate for inclusion.

| Figs. 1 through 3 | Same as specimens used for figures 5-7. |
| --- | --- |
| Fig. 4 | Biopsy of endoscopically examined Randall’s plaque from a 44 year old male with stagnant urine, ureter blocked with stone. |
| Fig. 5 | 71-year-old male with a 7cm incidentally discovered right renal mass who had a past medical history significant for arthritis, hypertension, hypothyroidism |
| Fig. 6 | 45-year-old female who underwent nephrectomy for chronic flank pain due to multiple failed repairs of ureteropelvic junction obstruction. She was dependent on a nephrostomy tube, did not have hydronephrosis, did have preserved split renal function and was not a candidate for ureteral reconstruction. The patient had a history of ipsilateral kidney stones in the distant past. |
| Fig. 7 | 70-year-old male who underwent laparoscopic right radical nephrectomy for an incidental renal mass. His past medical history was significant for gallstone pancreatitis, glaucoma, hyperlipidemia, gout, and hypertension. His prior surgeries included appendectomy, vasectomy, tonsillectomy, and eye surgery. His postoperative creatinine was 1.26 mg/dL |
| Figs. S1 and S2 | Same as Figure 5 |
| Fig. S3 | Same as Figure 5 |
| Fig. S4 | Same as Figure 6 |
| Fig. S5 | 41-year-old female with a large left renal stone who underwent percutaneous nephrolithotomy and the specimen was biopsied at this time. Past medical history was significant for anxiety, arthritis, blood clotting disorder, depression, arrhythmia, hypertension, herbal colon, kidney stones, pyelonephritis, thyroid disorder, and urinary tract infections. Past surgical history was significant for multiple cardiac ablations, national hysterectomy, and partial thyroidectomy. |
| Table S1 | Same as Figure 5 |

**SUPPLEMENTAL FIGURES**


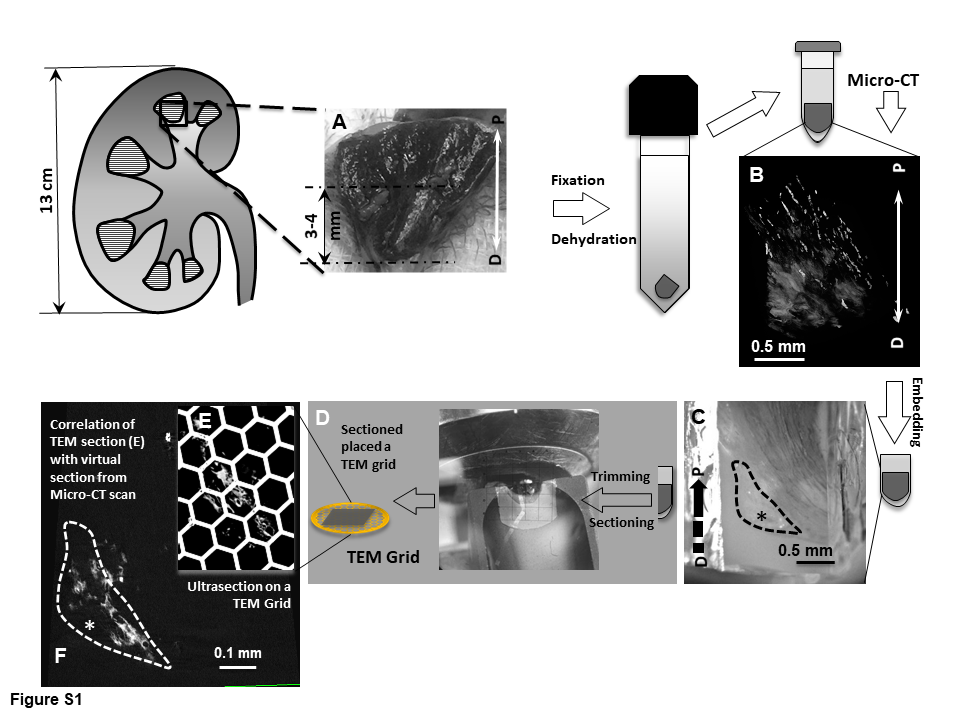


**Figure S1. Specimen preparation and work flow.** (A). **Human medullo-papillary complexes** dissected from surgically removed kidneys immediately placed into 100% ethanol for imaging using micro-XCT along with a reconstructed volume (B) are shown. (C) Subsequently, the specimen is embedded in epoxy, imaged using a light microscope and sectioned using an ultramicrotome. (D) Thin sections were collected on carbon/formvar-coated TEM grids. Correlative microscopy (asterisk) was performed between low resolution TEM (E) and high resolution micro-XCT tomograms (F), SEM of the surface of the ultrasectioned block to extract contextual information for regions of interest. D: Distal; P: Proximal

**Figure S2. Multiscale three-dimensional map illustrates anatomically-specific pathology.** This video illustrates the location of pathology in a human kidney to site-specific locations/regions 1 and 2 in the renal papilla within a human kidney.

**
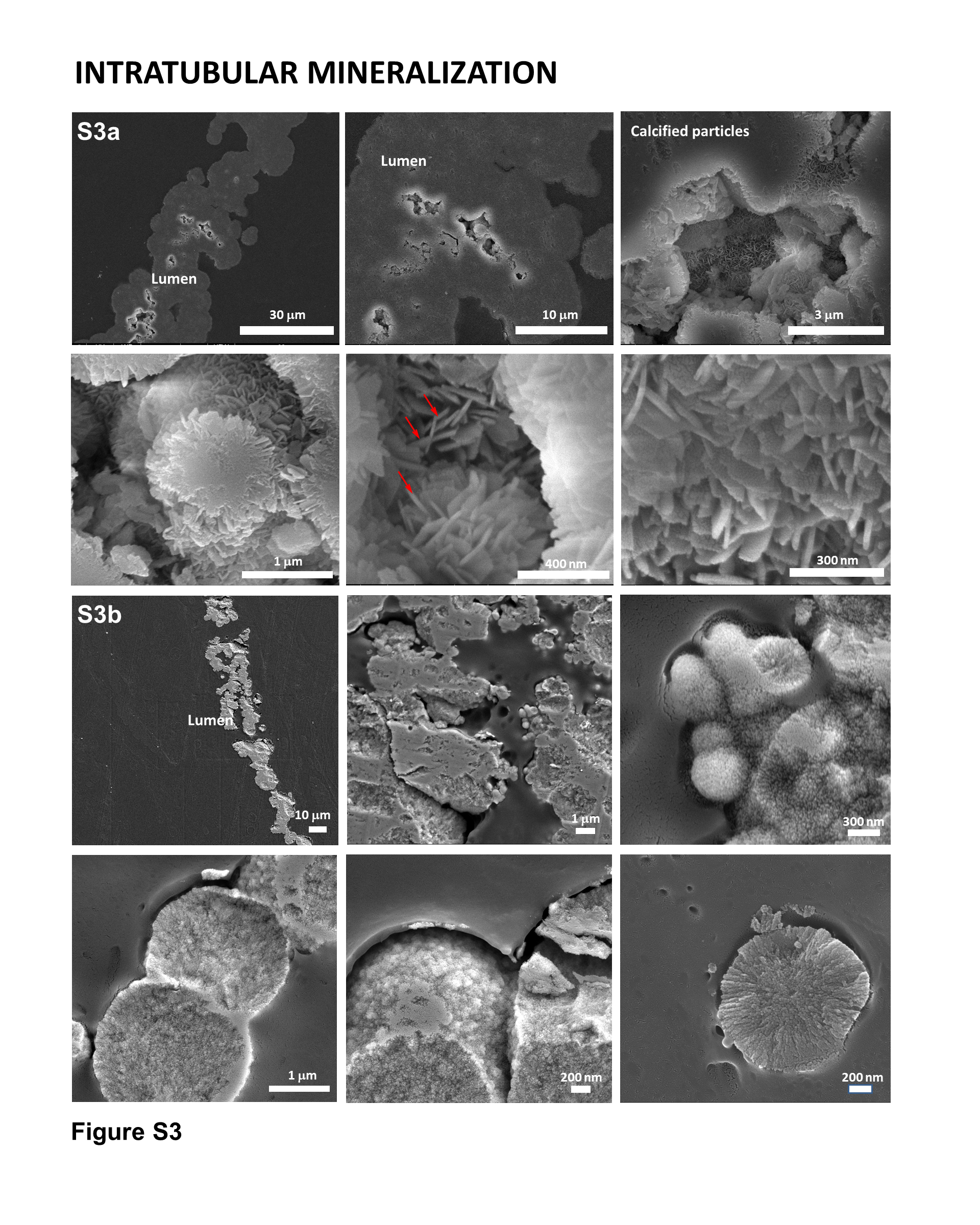

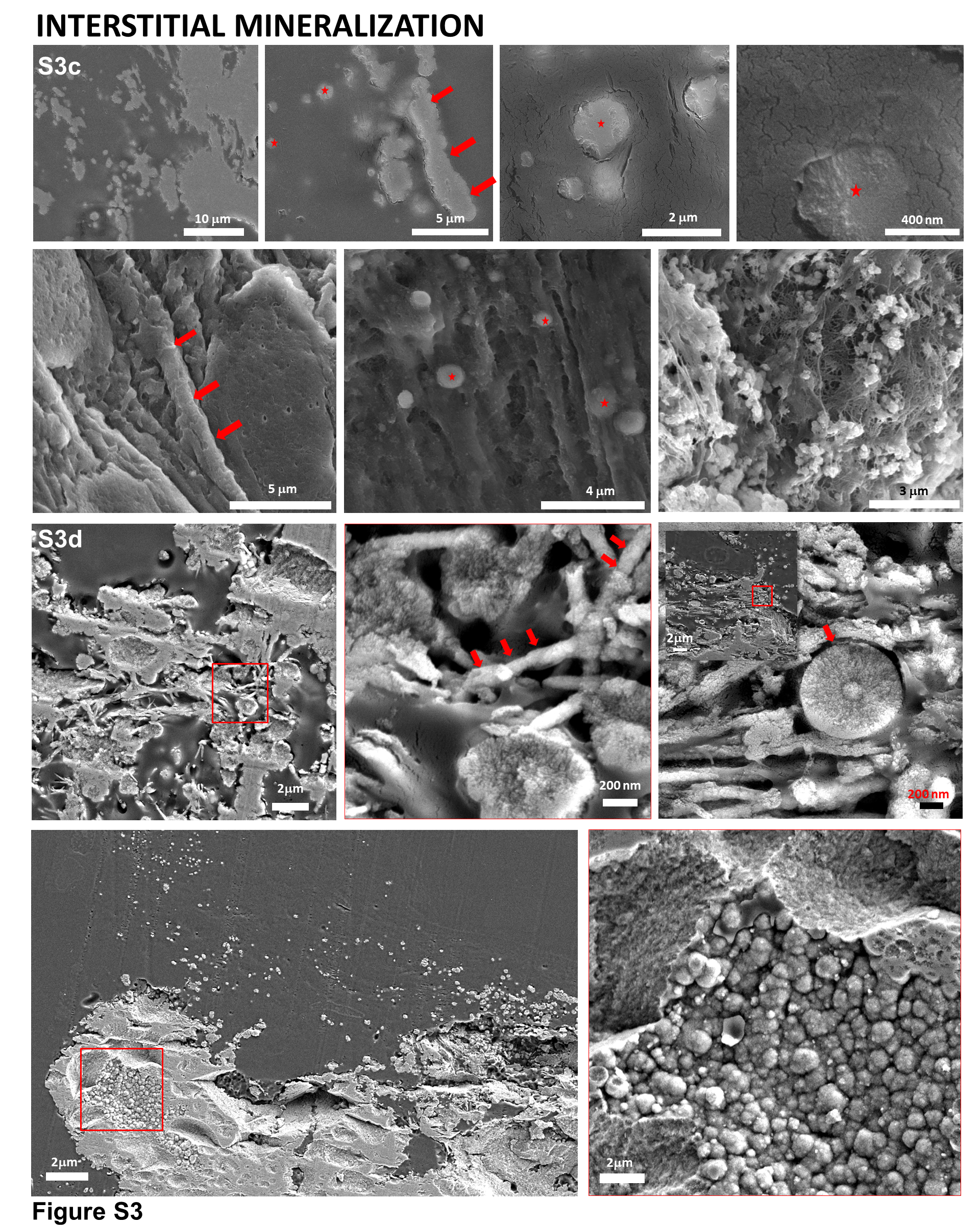
**

**Figure S3. Morphology of proximal intratubular (a, b) and distal interstitial (c, d) minerals.** (a and b) Low magnification scanning electron microscope (SEM) images of the trimmed surface show many voids within the tubule. Aggregates of plate-like crystals, but within circular cross sections are revealed in the high magnification SEM images. (c and d) Interstitial biominerals revealed numerous spherical nanostones. These specimens reveal nanostones (red stars) located in the interstitial regions rich in collagen fibrils (red arrows).

**
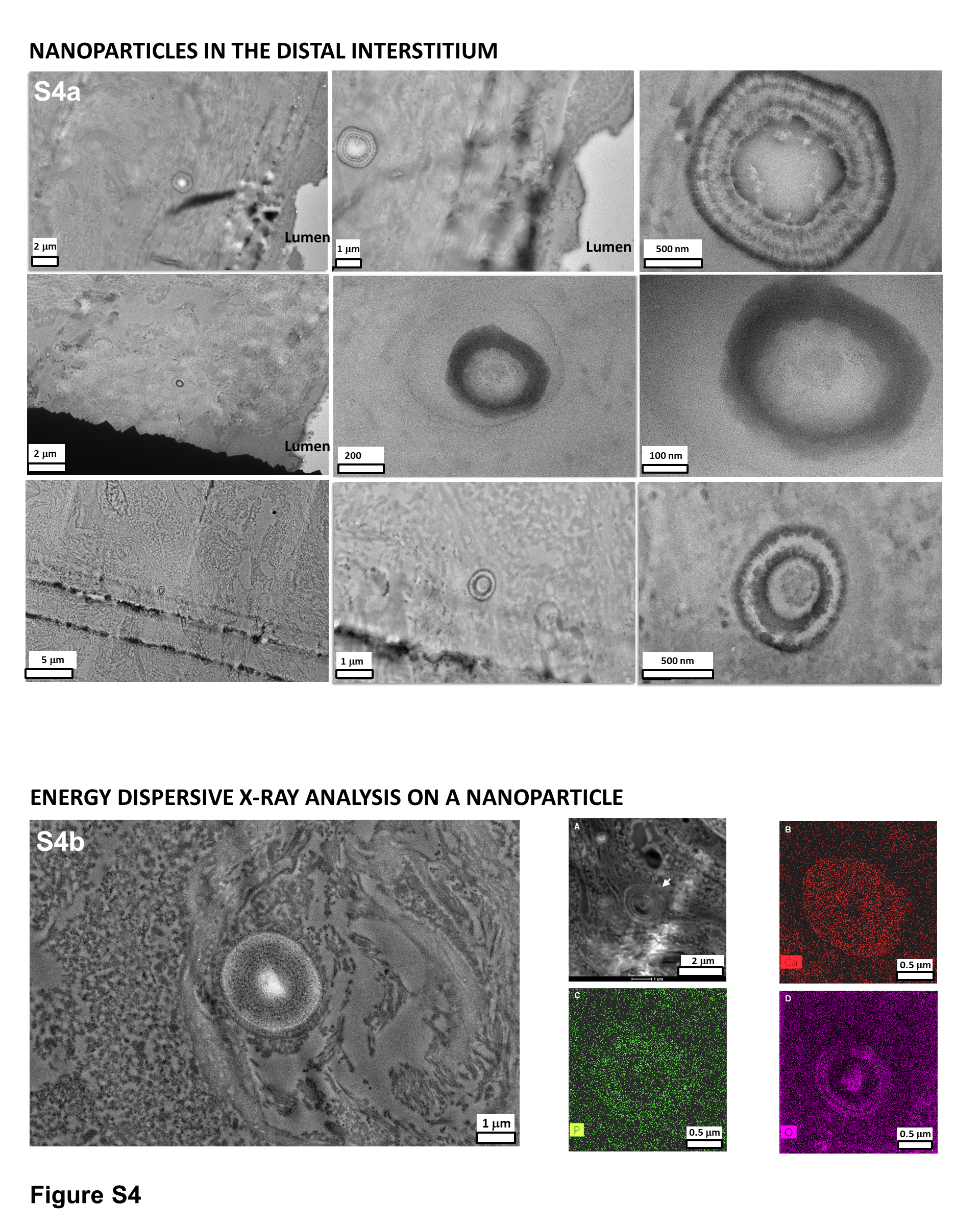
Figure S4.** **TEM and STEM micrographs of calcified nanoparticles within the distal interstitial matrix of a renal medullo-papillary complex.** (a). Morphological features of calcified nanoparticles within the distal interstitium of the MPC as visualized using a TEM. (b). Localization of calcium (Ca), phosphorus (P), and oxygen (O) were identified using energy dispersive spectroscopy within the calcified nanoparticles visualized using a scanning transmission electron microscope (structure).

**
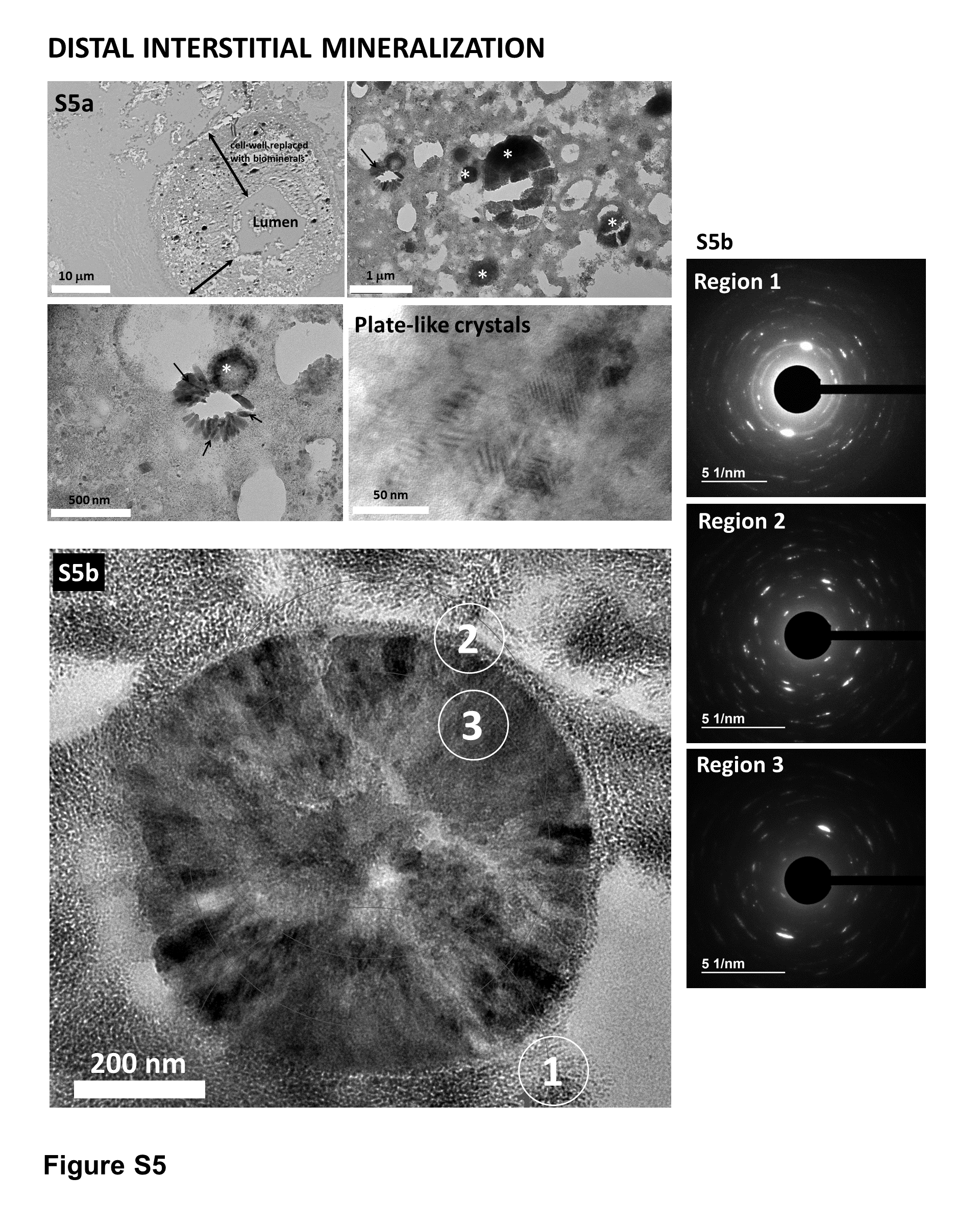
**

**Figure S5. TEM micrographs of distal interstitial mineralization.** (a). In the vicinity of a tubule, several nanostones with circular cross sections (asterisks) containing plate-like structures (arrows) were observed. (b). Note that the tubule wall has no intact cells. The selected area electron diffraction (SAED) patterns for 1-3 regions illustrated polycrystalline material although regions 1 and 3 indicated preferentially orientated crystals.

**
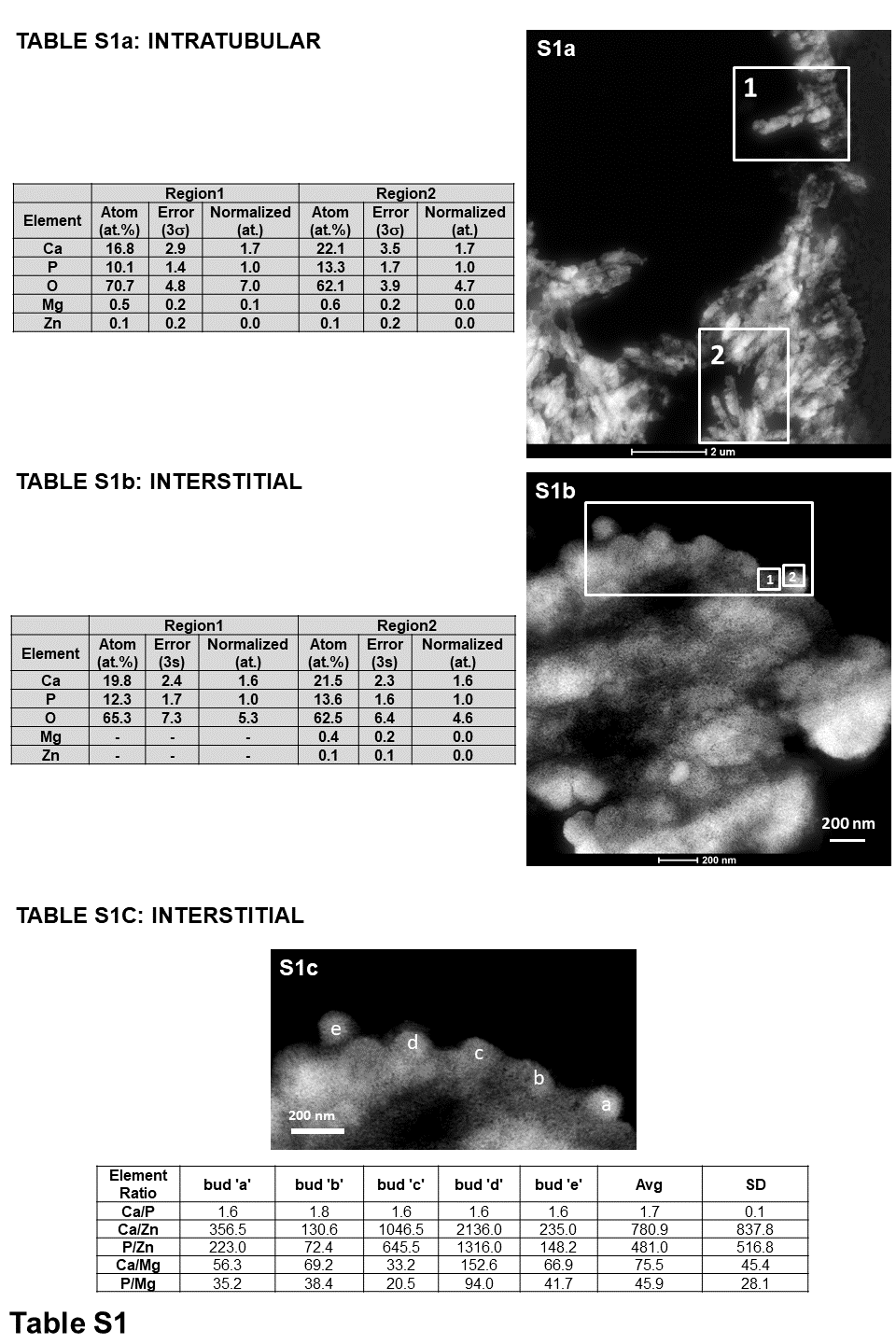
**

**Table S1. Quantitative analysis of the STEM-XEDS elemental maps for proximal intratubular and distal interstitial minerals.** (a). In regions 1 and 2 of Fig. 6a the calculated atomic percentage for Ca, P, O, Mg, and Zn are given with an error of three standard deviations (3σ) for each determined by the goodness of the peak fits. Atomic percent values normalized to the P concentration are also given. (b). Additional quantitative analysis of the STEM-XEDS elemental maps of a crystalline aggregate in interstitial mineralization from Fig. 6b. The mineral-front consisting of “buds” (a, b, c, d, e) used in the quantification are highlighted. No significant difference in chemical composition after T-test (P>0.5) was observed between the “buds”. **(c).** Quantitative analysis of the STEM-XEDS elemental maps for interstitial minerals. The calculated atomic percent for Ca, P, O, Mg, and Zn are given with an error of three standard deviations (3σ) for each determined by the goodness of the peak fits and the accuracy of the input cross sections for x-ray emission. Atomic percent values normalized to the P concentration are also given. Note: In order to ensure a robust analysis, only fits to the K-edge peaks (K-alpha and K-beta) were used for the quantification (i.e. the weak L-edge peaks below 1keV were not included).
